# Silicon and mechanical damage increase polyphenols and vitexin in *Passiflora incarnata* L.

**DOI:** 10.1038/s41598-021-01647-y

**Published:** 2021-11-11

**Authors:** Jonas Akenaton Venturineli Pagassini, Leandro José Grava de Godoy, Felipe Girotto Campos, Gustavo Ribeiro Barzotto, Maria Aparecida Ribeiro Vieira, Carmen Sílvia Fernandes Boaro

**Affiliations:** 1 School of Agriculture, São Paulo State University, Botucatu, Brazil; 2 Campus of Registro, São Paulo State University, Registro, Brazil; 3 Institute of Biosciences, São Paulo State University, Botucatu, Brazil

**Keywords:** Photosynthesis, Plant physiology, Plant signalling, Plant stress responses, Secondary metabolism

## Abstract

*Passiflora incarnata* L. is a species of global pharmacological importance, has not been fully studied in the context of cultivation and management. It is known that silicon acts on abiotic stress and promotes phenols synthesis. The practice of mechanical damage is widely used in *P. incarnata* crops, and its interaction with silicon can have a significant influence on plant metabolism. Therefore, our objective was to investigate the effects of silicon and mechanical damage on photosynthesis, polyphenols and vitexin of *P. incarnata*. The experiment was conducted in a factorial design with SiO_2_ concentrations (0, 1, 2, 3 mM) and presence or absence of mechanical damage. It was found that mechanical damage improved photosynthetic performance at lower concentrations or absence of silicon. Moreover, this condition promoted an increasing in vitexin concentration when SiO_2_ was not provided. The application of 3 mM Si is recommended to increase polyphenols and vitexin, without harming dry mass of aerial part. The interaction between silicon and mechanical damage could be a tool to increase agronomic yield and commercial value of the *P. incarnata* crop.

## Introduction

*Passiflora incarnata* L. is a perennial tropical plant with a climbing herbaceous habit, whose center of origin is in the southeastern United States^1–3^, being also cultivated in other countries of the American continent, Europe, Asia, Africa and Australia. It is studied in the pharmacological field because presents chemical substances of interest to health and is a component of anxiolytic drugs^4–7^.

The species is part of the United States Pharmacopeia (U.S.P) and European Pharmacopoeia and it is cataloged in the National List of Medicinal Plants of Interest to the Unified Health System (RENISUS list—Brazil)^8^.

*Passiflora incarnata* has medicinal potential for social impact, with neurogenic action and beneficial to functions related to memory and learning^6^. Regarding this potential, vitexin is a flavone that presents antioxidant, cardioprotective, anticarcinogenic, anti-inflammatory, antidiabetic, and anticonvulsant actions^9–13^. Diseases related to the nervous system have become increasingly common, with increased occurrences of depression and anxiety^13,14^, which suggests that researches with this species should be expanded.

While recent studies address the cultivation, nutrition and management of *P*. *incarnata*^15–18^ few studies have focused on increasing bioactive molecules synthesis. Beneficial elements, such as silicon (Si), are an alternative for a better performance of agricultural crops and their effects are observed in plants subjected to stress, presenting better photosynthetic performance, greater growth and phenolic compounds accumulation. These effects are explained by the greater activity of antioxidant enzymes and the phenylalanine ammonia-lyase (PAL), which acts in the pathway of phenols and other molecules with medicinal importance^19–22^.

Si can act as a signal for the genes expression in the photochemical stage and for other genes that activate enzymes, participating in the polyphenols and flavonoids synthesis^19–22^. In addition, the element can be complexed with phenolic compounds, making them insoluble, expanding their mobility in the apoplast due to the transpiration flow and also stimulating the biosynthesis of these compounds, based on the genes expression from the PAL pathway, promoted by higher Si concentrations^23,24^.

Beneficial effects of Si have already been reported for *Passiflora edulis* Sims.^25,26^, who verified its deposition in the cell wall epicuticle, besides the increase in stomatal conductance, CO_2_ assimilation rate, transpiration rate, dry mass of leaves and root. However, the higher Si concentration used in the mentioned study reduced the chlorophyll concentration, which indicates that the element concentration is crucial to obtain beneficial results.

In the commercial crops of *P. incarnata* more than one harvest is expected, enabling a continuous supply of leaves and stems to the pharmaceutical production chain^27^. The complete harvesting of the aerial part promotes mechanical damage, which can signal the production of phenolic compounds, since this stress may be influence the activity of the PAL enzyme and other enzymes in the polyphenol pathway^28^**.**

The Si supply and mechanical damage can result in an increase in biomass and active molecules, contributing to the production chain of the species. The objective of this study was to investigate the Si and mechanical damage effects on photosynthetic metabolism and on the polyphenols and vitexin synthesis in *P. incarnata*.

## Results

### Chlorophyll *a* fluorescence and gas exchange

**Figure 1:**
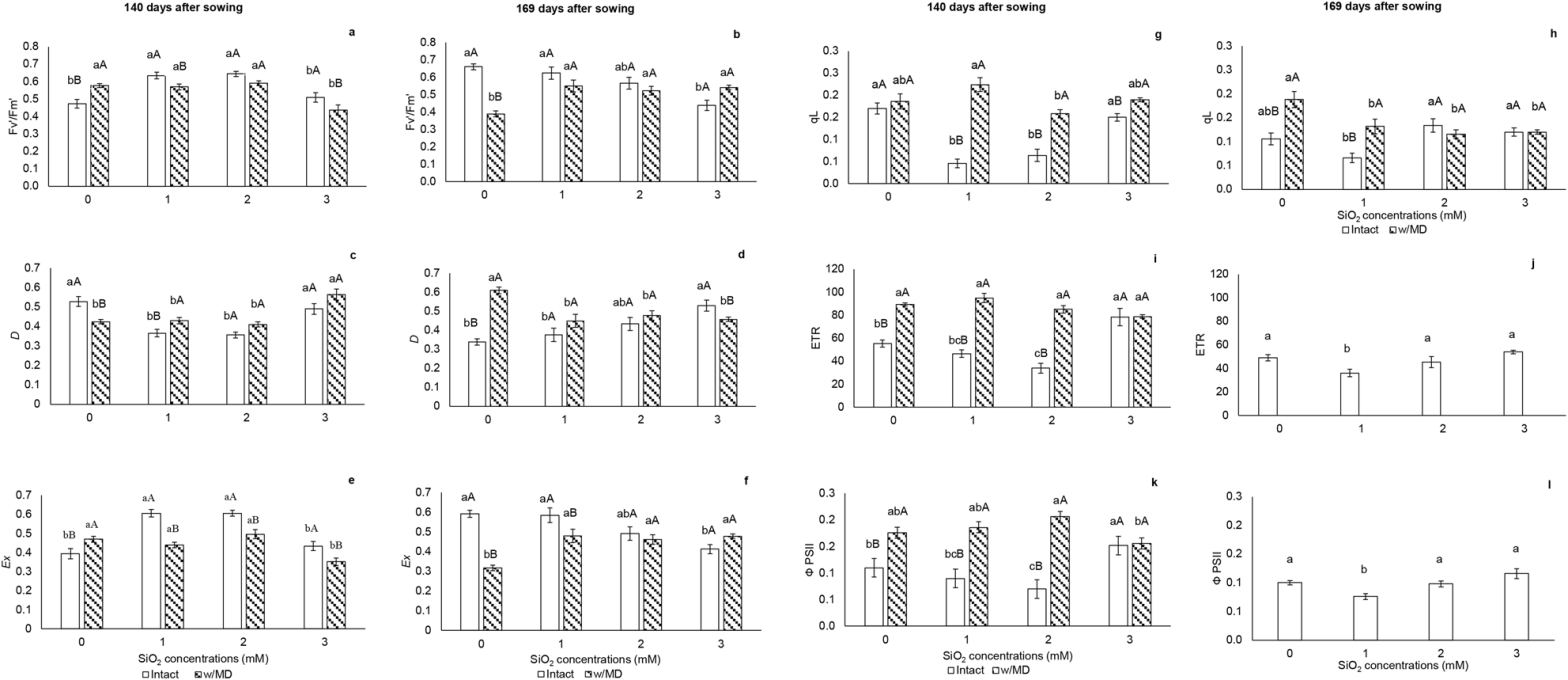
Chlorophyll *a* fluorescence. (**a**,**b**) Potential quantum efficiency of open PSII center (Fv′/Fm′) p < 0.01; (**c**,**d**) energy fraction absorbed by PSII antenna that is dissipated as heat (*D*) p < 0.01; (**e**,**f**) energy fraction not dissipated in the antenna that cannot be used for photochemistry stage (*Ex*) p < 0.01; (**g**,**h**) photochemical quenching (qL) p < 0.01; (**i**,**j**) electron transport rate (ETR) p < 0.01, (**k**,**l**) effective quantum efficiency of photosystem II (ΦPSII) p < 0.01 in *Passiflora incarnata* L. with mechanical damage (w/MD) and without mechanical damage (intact), subjected to SiO_2_ variations at 140 and 169 days after sowing. Values corresponding to the averages ± SE. Capital letters compare plants with and without mechanical damage and lower letters compare SiO_2_ variations. ETR and PSII results were significant only for SiO_2_ variations. Other results were significant for the interaction between SiO_2_ variations and the presence/absence of mechanical damage.

In the absence of silicon, the potential quantum efficiency of the open reaction center (Fv′/Fm′) at 140 days after sowing (DAS) was higher in plants that received mechanical damage. In the absence of damage, at 169 DAS, Fv′/Fm′ was lower in plants grown with 3 mM SiO_2_ (Fig. 1a,b).

At 140 DAS, plants that received mechanical damage and cultivated with 1 and 2 mM SiO_2_ showed higher photosystem performance, represented by photochemical quenching (qL), electron transport rate (ETR) and effective quantum efficiency of photosystem II (ФPSII) than intact plants at the same concentrations (Fig. 1g,i,k). In the absence of mechanical damage, energy fraction absorbed by PSII antenna that is dissipated as heat (*D*) was higher and energy not dissipated and not used for the photochemical phase (*Ex*) was lower in plants subjected to 0 and 3 mM SiO_2_, which may indicate photoprotection (Fig. 1c,e).

At 169 DAS, regardless the damage, plants cultivated at concentration 1 mM SiO_2_ showed lower ETR and ФPSII (Fig. 1j,l). Among plants that did not receive Si, those that received mechanical damage had higher *D* and qL and lower *Ex* compared to intact plants (Fig. 1d,f,h).

**Figure 2:**
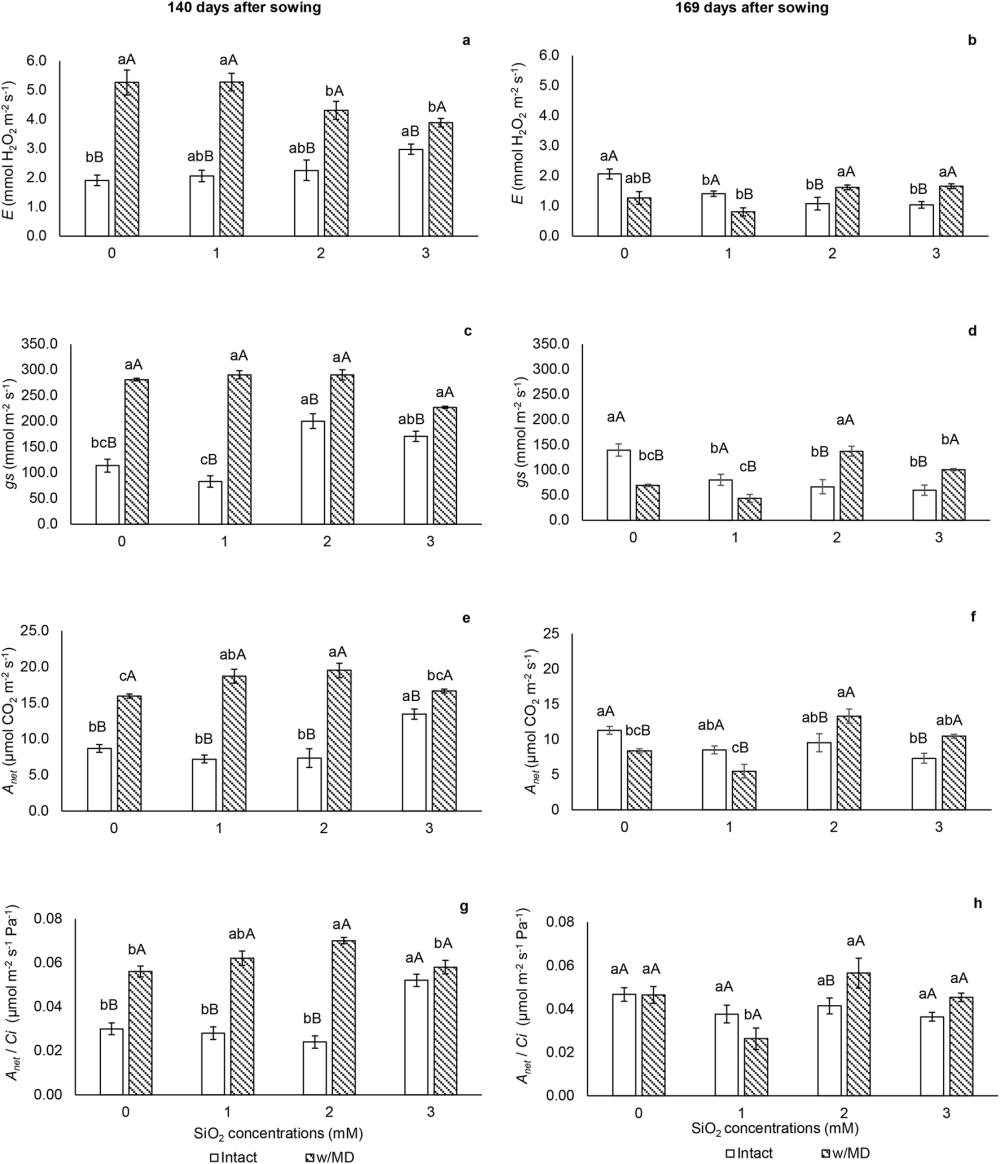
Gas exchange. (**a**,**b**)Transpiration rate (*E,* mmol H_2_O m^−2^ s^−1^) p < 0.01; (**c**,**d**) stomatal conductance (*g*_*s*_, mmol m^−2^ s^−1^) p < 0.01; (**e**,**f**) CO_2_ assimilation rate (*A*_*net*_, μmol CO_2_ m^−2^ s^−1^) p < 0.01; (**g**,**h**) RuBisCO carboxylation efficiency (*A*_*net*_/*C*_*i*_*,* μmol m^−2^ s^−1^ Pa^−1^) p < 0.03 in *Passiflora incarnata* L. with mechanical damage (w/MD) and without mechanical damage (intact), subjected to SiO_2_ variations at 140 and 169 days after sowing. Values corresponding to the averages ± SE. Capital letters compare plants with and without mechanical damage and lower letters compare SiO_2_ variations.

At 140 DAS plants subjected to damage had higher transpiration rate (*E*) regardless the SiO_2_ level (Fig. 2a). At 169 DAS, plants with 2 and 3 mM SiO_2_ with mechanical damage showed high transpiration rate. Among intact plants, those with Si had a lower *E* (Fig. 2b).

Stomatal conductance (*g*_*s*_), CO_2_ assimilation rate (*A*_*net*_) and RuBisCO carboxylation efficiency (*A*_*net*_/*C*_*i*_) were higher at 140 DAS in plants subjected to mechanical damage, except for plants grown with 3 mM SiO_2_ (Fig. 2c,e,g). At 169 DAS, *g*_*s*_*, A*_*net*_ and *A*_*net*_/*C*_*i*_ were higher in plants with damage at the highest SiO_2_ concentrations (Fig. 2d,f,h).

### Hydrogen peroxide and lipid peroxidation

**Figure 3:**
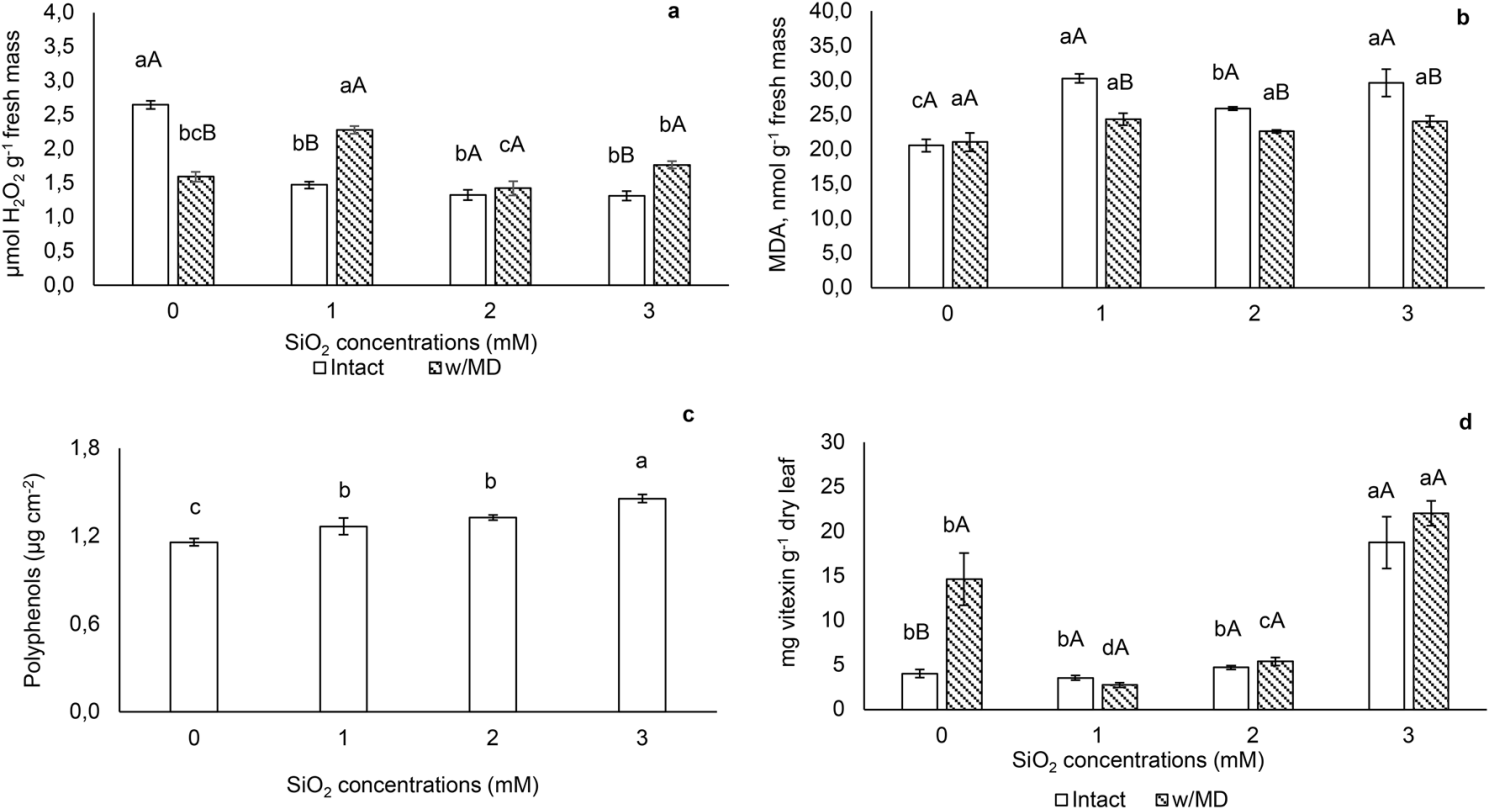
(**a**) Hydrogen peroxide, (**b**) lipid peroxidation, (**c**) polyphenols and (**d**) vitexin concentration. H_2_O_2_ p < 0.05; lipid peroxidation expressed by the formation of malonaldehyde (MDA) p < 0.05; polyphenols p < 0.01; vitexin p < 0.01 in *Passiflora incarnata* L. with mechanical damage (w/MD) and without mechanical damage (intact), subjected to SiO_2_ variations. Values corresponding to the averages ± SE. Capital letters compare plants with and without mechanical damage and lower letters compare SiO_2_ variations. Polyphenols results were significant only for SiO_2_ variations. Other results were significant for the interaction between SiO_2_ variations and the presence/absence of mechanical damage.

Plants with 1 mM SiO_2_ showed a higher concentration of hydrogen peroxide (H_2_O_2)_ when damage occurred. In intact plants, SiO_2_ supply reduced hydrogen peroxide, except for the 2 mM SiO_2_ concentration. (Fig. 3a). In the mechanical damage absence, lipid peroxidation, presented as malondialdehyde concentration (MDA) was higher in plants grown with SiO_2_ and in the presence of mechanical damage, there was no difference between plants (Fig. 3b).

### Polyphenol index and vitexin

Higher polyphenol content was revealed by plants grown with 3 mM SiO_2_ and plants grown with 1 and 2 mM SiO_2_ showed intermediate concentrations, not differing from each other. In this evaluation, there was no significant effect of mechanical damage or even its interaction with SiO_2_ levels (Fig. 3c).

**Figure 4:**
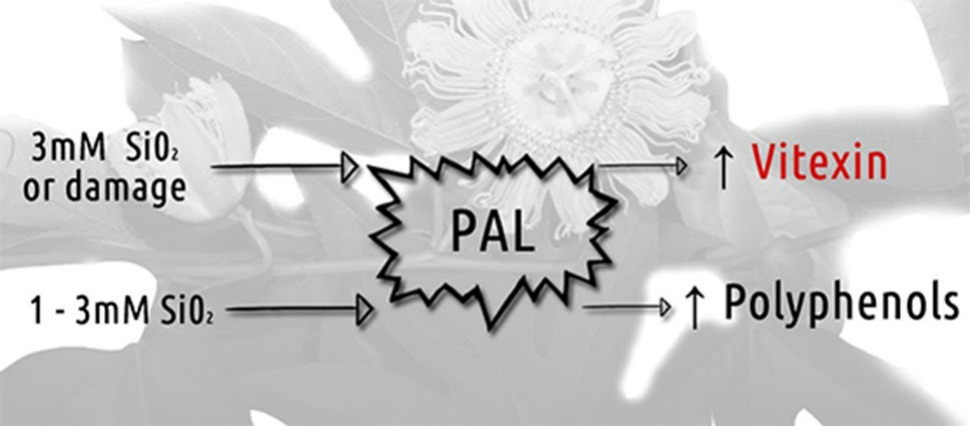
Simplified scheme of silicon action and mechanical damage in *Passiflora incarnata* L., in increasing the activity of the enzyme phenylalanine ammonia-lyase (PAL), resulting in an increase in polyphenols and vitexin^23^.

Plants collected at 169 DAS, regardless the mechanical damage, had the highest vitexin content with 3 mM SiO_2_ (Fig. 3d). Plants grown without Si and with mechanical damage showed a higher content of vitexin when compared to intact plants (Figs. 3d and 4).

### Carbohydrates

**Figure 5:**
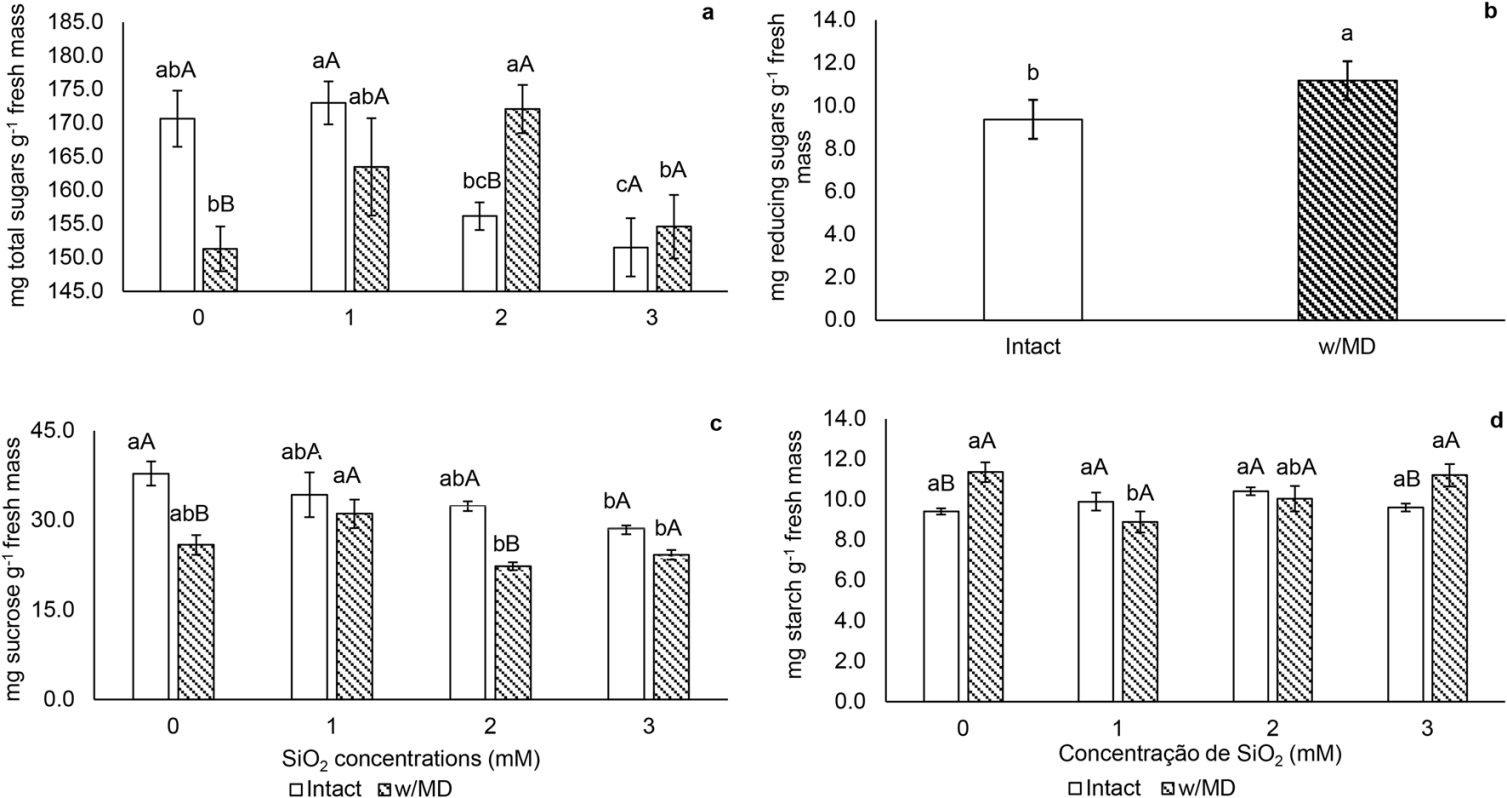
Carbohydrates. (**a**) Total sugars (p < 0.05); (**b**) reducing sugars (p < 0.04); (**c**) sucrose (p < 0.04); (**d**) starch (p < 0.05) in *Passiflora incarnata* L. with mechanical damage (w/MD) and without mechanical damage (intact), subjected to SiO_2_ variations. Values corresponding to the averages ± SE. Capital letters compare plants with and without mechanical damage and lower letters compare SiO_2_ variations. Reducing sugars results were significant only for mechanical damage presence/absence. Other results were significant for the interaction between SiO_2_ variations and the presence/absence of mechanical damage.

Plants without silicon subjected to mechanical damage showed investment in reserve carbohydrates, such as starch, while intact plants showed high total soluble sugars concentrations (Fig. 5a,d).

Plants with mechanical damage showed a high amount of total soluble sugars when grown with 2 mM SiO_2_. Intact plants showed a lower amount of total soluble sugars with 3 mM SiO_2_ (Fig. 5a). Plants subjected to mechanical damage showed a higher amount of reducing sugars, regardless the SiO_2_ concentration (Fig. 5b).

Intact plants with 3 mM SiO_2_ showed lower sucrose concentration when compared to intact plants without Si. In plants with mechanical damage, the highest concentration of sucrose was found in plants with 1 mM SiO_2_, which did not differ from plants without Si (Fig. 5c).

Among plants cultivated without Si and with 3 mM SiO_2_, those with mechanical damage showed higher starch accumulation. In general, plants grown with 1 mM SiO_2,_ and damage showed less starch accumulation. In the intact plants, the starch concentration did not differ (Fig. 5d).

### Growth indices

**Figure 6:**
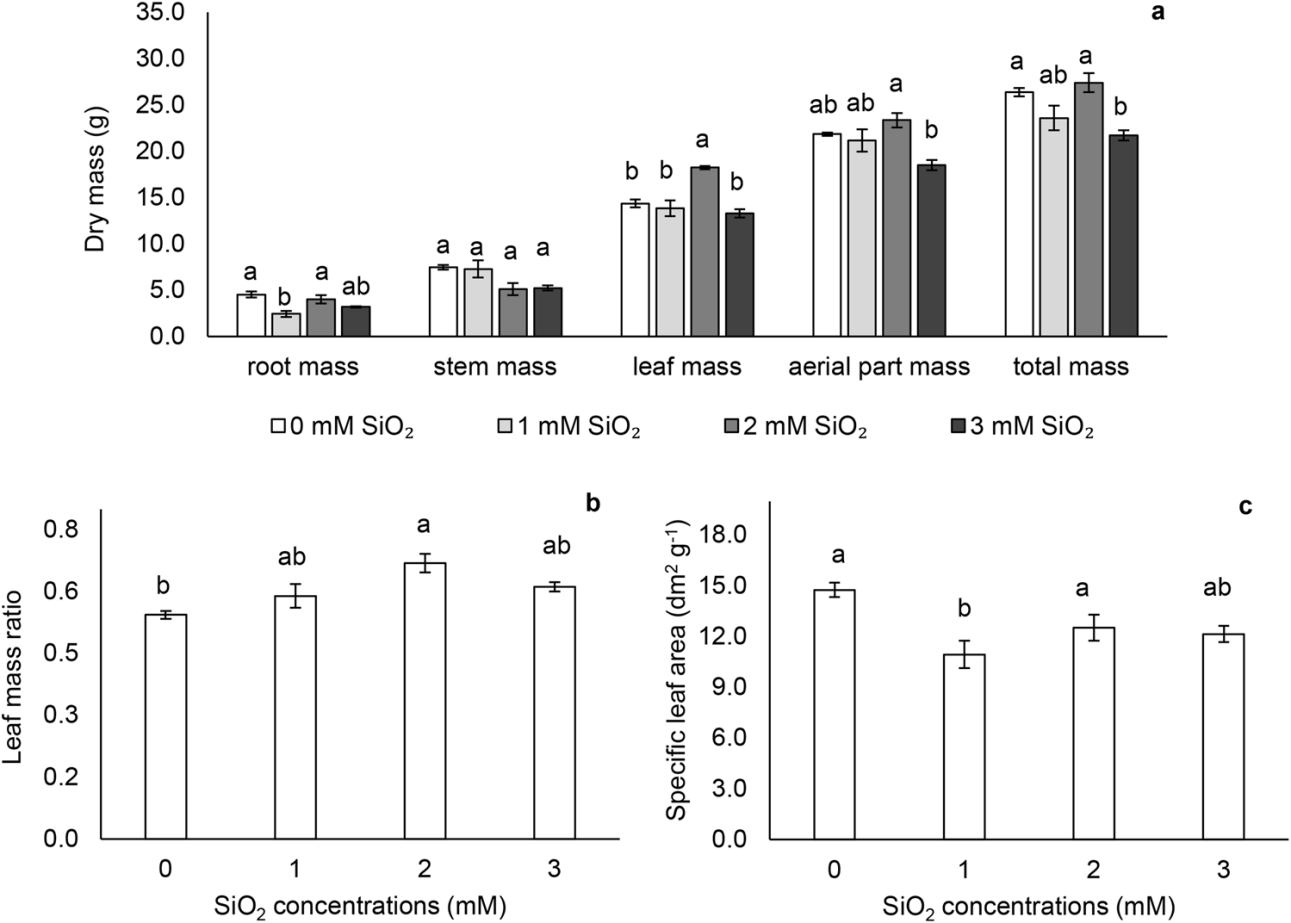
Biomass. (**a**) Dry masses of roots (p < 0.01), stems (p < 0.03), leaves (p < 0.01), aerial part (p < 0.01) and total plant (p < 0.01); (**b**) leaf mass ratio (p < 0.01); (**c**) specific leaf area (p < 0.01) in *Passiflora incarnata* subjected to SiO_2_ variations. Values corresponding to the averages ± SE.

Plants cultivated with 2 mM SiO_2_ showed higher dry mass of leaves (LDM) and total (TDM), highlighting that in TDM, plants with 2 mM SiO_2_ did not differ from those with lower concentration or Si absence. When 3 mM SiO_2_ was provided, TDM was reduced (Fig. 6a). The leaf mass ratio (LMR) was higher in plants that received 2 mM SiO_2_, not different from plants grown with 1 and 3 mM SiO_2_ (Fig. 6b).

The LMR expresses the plant area useful for photosynthesis, resulting in plant mass, and the specific leaf area (SLA) data reveal area per leaf mass, indicating its thickness (Fig. 6c). The concentration of 2 mM SiO_2_ increased the LMR and 1 mM SiO_2_ decreased SLA compared to control plants.

### Leaf silicon content

**Figure 7:**
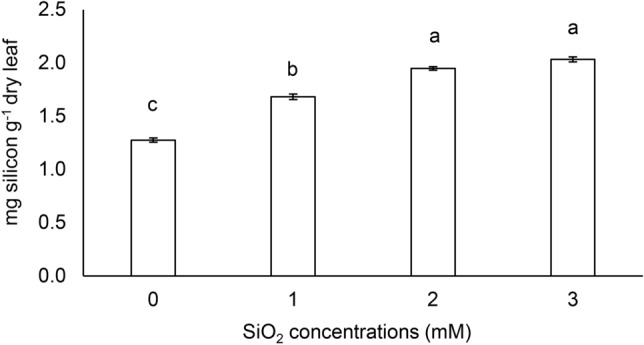
Silicon content in leaves from *Passiflora incarnata* L. subjected to SiO_2_ variations. Values corresponding to the averages ± SE.

The supply of SiO_2_ increased the Si content in the leaves. When 1 mM SiO_2_ was supplied, Si concentration in the leaves was intermediate and the highest content was found in plants grown with 2 and 3 mM SiO_2_ (Fig. 7).

These results indicate that the source of Si used for the conditions of this study was adequate. Si was quickly translocated to the leaves, since SiO_2_ supply occurred at 124 DAS and leaf collect, which resulted in biochemical evaluations, was performed at 169 DAS (Fig. 7).

### Heatmap

**Figure 8:**
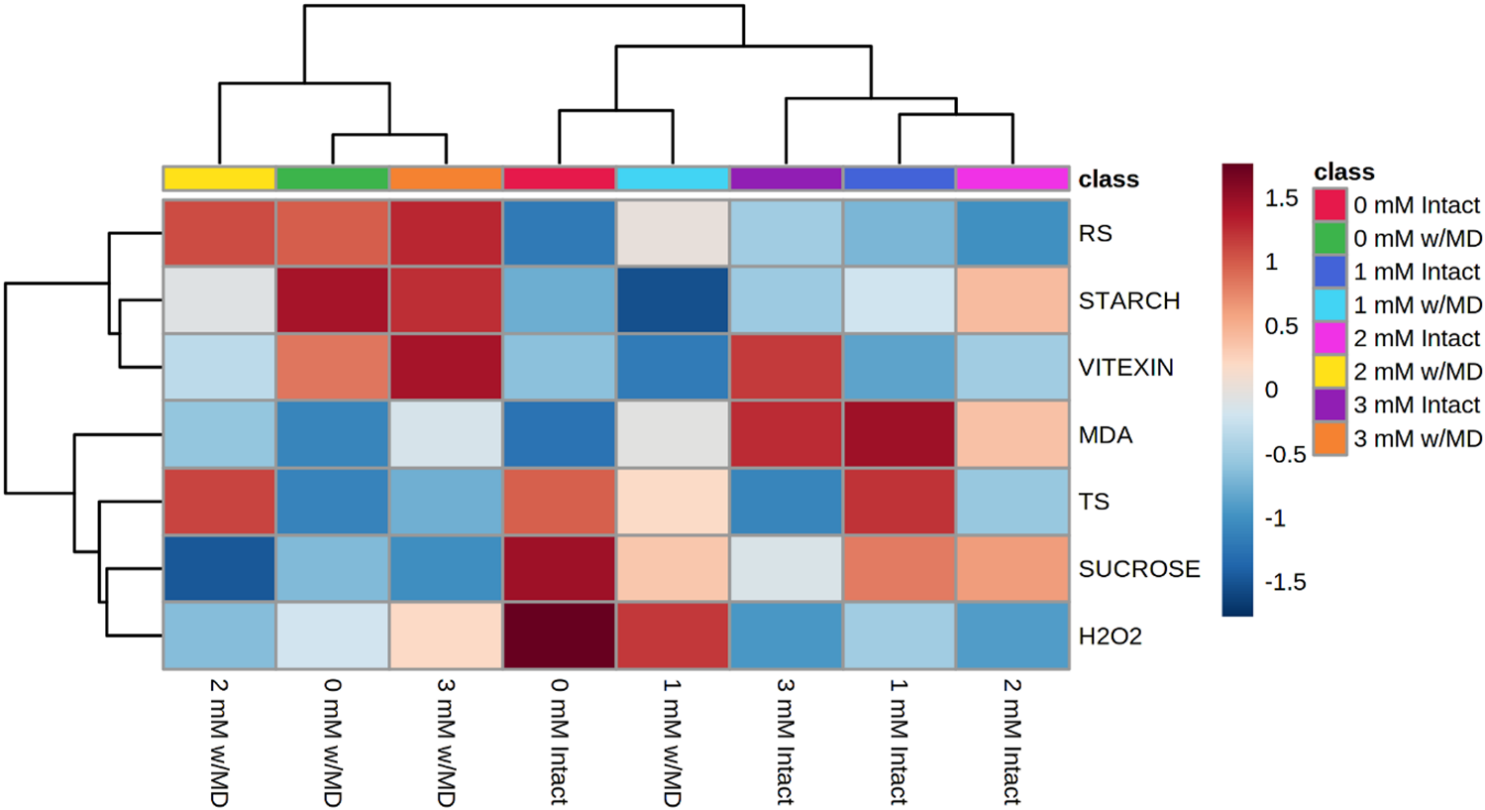
Heatmap. Hierarchical cluster analysis presented as a heatmap on evaluations of reducing sugars (RS), starch, vitexin, lipid peroxidation expressed by the formation of malonaldehyde (MDA), total sugars (TS), sucrose and hydrogen peroxide (H_2_O_2_) in *Passiflora incarnata* L. with mechanical damage (w/MD) and without mechanical damage (intact), subjected to SiO_2_ variations (0, 1, 2 and 3 mM). Software used to create this figure was MetaboAnalyst (v4.0, https://www.metaboanalyst.ca/).

A heatmap was drawn up to demonstrate the similarity between treatments and the correlation between biochemical variables (Fig. 8). It is possible to observe the formation of two groups in which the treatments in each group have similarity for the variables. The first group consisted of treatments with damage and 0, 2 and 3 mM SiO_2_. The second group consisted of treatments with intact plants and plants with damage and 1 mM SiO_2_. The treatments in the first group showed the highest averages (red squares) for the variables reducing sugars and starch, and the lowest for sucrose and MDA. In this group, treatments 0 and 3 mM SiO_2_ with damage had the highest averages for vitexin. On the other hand, the treatments in the second group had the lowest averages for reducing sugars and high for sucrose. In this group, the intact plants treatments that received Si had higher MDA averages. We highlight in this group the treatment with 3 mM SiO_2_ which presented the highest vitexin average, opposite to the others, which indicates a relationship with the SiO_2_ level supplied. When 3 mM SiO_2_ were used in plants with or without damage, higher vitexin averages were verified. However, intact plants with 3 mM SiO_2_ revealed high MDA concentration.

## Discussion

Our study results emphasize the Si action on the metabolism of plants subjected to abiotic stress, providing better performance under adverse conditions, as observed in other studies^29^.

The mechanical damage in *P. incarnata* at 140 DAS stimulated remained bud’s photosynthetic activity, suggesting compensatory photosynthesis^30^, since the removal of old branches allows interception of solar radiation better by young branches. The requirement of higher demand for photoassimilates by new tissues, can stimulate the development and photochemical activity, enabling increased electron flow^31,32^. It’s responsible for higher production of reducing agents used in carbon assimilation, observed in the present study, respectively, by higher ETR and ФPSII.

*P*. *incarnata* plants with 1 and 2 mM SiO_2_ and mechanical damage were efficient in overcoming this damage and restoring themselves, which is observed in the high photochemical efficiency at 140 DAS and high *A*_*net*_*/Ci, A*_*net*_ and *g*_*s.*_ At 169 DAS the supply of 2 mM SiO_2_ promoted increase of *g*_*s*_ and *A*_*net*_*.* These results are in agreement with those verified in the literature^25,26^, in which data of increase in *g*_*s*_, *A*_*net*_, *E* and dry mass of leaves are verified in *P. edulis* when Si was supplied.

According to the study by Zhang et al.^22^, the supply of Si may have promoted greater expression of the genes *PetE*, *PetF*, *PsbP*, *PsbQ*, *PsbW* and *Psb*, which are important for the photochemical step of photosynthesis. Gene expression may have contributed to the production of reducing agents used in the biochemical stage of photosynthesis, as indicated in other studies^29,33^, and observed in the high qL at 140 DAS, presented in this study.

At 169 DAS the mechanical damage was preponderant to maintain photochemical energy’s direction for the production of reducing agents, since the highest qL was observed in plants without Si. The mechanical damage may favor photochemical activity increase, stimulated by high nitrogen demand for new tissues formation, as well as higher incident radiation stimulates nitrate absorption by the roots. Besides, the reduction of nitrate occurs mainly in leaves, as it is a strong electron drain, it can stimulate greater photochemical activity^31^. It is noteworthy that nitrogen source used in this work was mainly nitric.

The higher photosynthetic activity, reflected by *A*_*net,*_* g*_*s*_ and *A*_*net*_/*C*_*i*_, may have contributed to a high concentration of total and reducing sugars, directing resources for growth, biomass accumulation and lower MDA accumulation, a result that indicates low stress in plants grown with 2 mM SiO_2_ and mechanical damage. The Si supply in plants with different stress modalities promotes an increase in the activity of antioxidant enzymes, which neutralize reactive oxygen species, decreasing lipid peroxidation^34–36^.

Si supply was effective in signaling polyphenol synthesis, as described in the literature^23,24^. We highlight the increase in vitexin provided by the higher dose of Si supplied to *P. incarnata*. Si promotes greater activity of the PAL enzyme, which participates in the phenols and flavonoids synthesis^23,24^.

Potassium silicate (5, 7.5 and 10 mM) influences apigenin^19^, a precursor flavone of vitexin, which may explain, in this study, the accumulation of vitexin in *P*. *incarnata* cultivated with 3 mM SiO_2._ The signaling for vitexin production is dependent on Si concentrations and seems not to be related to higher lipid peroxidation and activation of the enzymatic antioxidant system. Mechanical stress can also influence the activity of the PAL enzyme and other enzymes in the polyphenol pathway, as suggested by the results of Liu et al.^28^, and confirmed in this study in the control treatment with absence of Si and with w/MD.

In the presence of mechanical damage, plants grown with 1 and 2 mM SiO_2_ were efficient in overcoming stress and these concentrations contributed to the synthesis of polyphenols. It is suggested that these concentrations were enough to signal the PAL metabolic pathway, which promoted an increase in the polyphenol index. As observed in the evaluation of vitexin, the increased activity of the PAL enzyme is stimulated by the supply of Si, resulting in an increase in the content of other phenolic compounds, as related in other studies^23,24^.

Damaged plants accumulated more starch than intact ones in the absence of Si. Starch may have been the result of mechanical stress, activating the enzymatic antioxidant system that reduced free H_2_O_2_. Among plants that didn’t receive Si, the stress that resulted in the accumulation of starch may be related to a higher content of vitexin, since stored starch may act as source of carbohydrates for the development of new tissues, in addition to providing carbon skeletons for flavonoid synthesis. Results by Castrillón-Arbeláez et al.^37^ reveal that mechanical damage is related to the expression of starch synthase, demonstrating an increase in this carbohydrate. In plants with Si, the accumulation of vitexin should not be related to starch resulting from stress, but the possible signaling triggered by the higher dose of Si supplied, acting on vitexin precursors^19^.

Among plants that received 3 mM SiO_2_, the absence of difference in total soluble sugars, reducing sugars and sucrose may indicate that the production of carbon skeletons was not altered. The starch concentration in plants with 3 mM SiO_2_ and mechanical damage suggests accumulation to overcome stress, similar to that observed in plants without Si and with mechanical damage. The supply of 1 mM SiO_2_ in plants with mechanical damage increased H_2_O_2_ concentration in leaves, but did not result in higher MDA. Plants with damage and Si also had lower MDA than intact plants with Si, indicating the supply of Si under stress conditions contributes to the efficiency of the enzymatic antioxidant system^29^.

The Si supplied to intact plants resulted in an increase in lipid peroxidation, although a higher free H_2_O_2_ content was not detected, also pointed out by Coskun et al.^29^. Only the 3 mM SiO_2_ concentration was effective in increasing the vitexin content. The results observed with Si supply in intact plants indicate that the stress demonstrated by the higher MDA seems not to be related to the higher vitexin synthesis, which suggests another signaling pathway.

We discovered that *P*. *incarnata* showed greater photosynthetic performance when subjected to mechanical damage, which may have triggered a signaling cascade and, associated with Si, resulted in less MDA, with damage recovery and accumulation of phenolic compounds. At a concentration equal to 3 mM SiO_2_, there was higher vitexin accumulation in the plants and a lower dry mass than other treatments. At low Si concentrations, the photosynthetic performance suggests overcoming the mechanical damage.

In *P*. *incarnata* crops, mechanical damage is performed by removing the aerial part, which can lead to an increase in vitexin production. The application of 3 mM Si is recommended to increase polyphenols and vitexin, without harming dry mass of aerial part. Supplying 3 mM SiO_2_ with increased vitexin by 150% and polyphenols by 130%, suggesting the potential of Si in the phenolic compounds increase in plants^23,24^, which may be important in the herbal medicines development for the treatment of diseases related to the central nervous system^9,38^. Thus, the interaction between silicon and mechanical damage could be a tool to increase agronomic yield and commercial value of the *P. incarnata* crop.

## Methods

### Experimental conditions

The experiment was carried out at the Department of Biostatistics, Plant Biology, Parasitology and Zoology at São Paulo State University (UNESP), Botucatu (São Paulo, Brazil), geographic coordinates 22° 53′ 09″W 48° 26′ 42″ S and 800 m average altitude, in a *Van der Hoeven* greenhouse type with pad fan and temperature about 25 °C ± 5.

### Plant material

Certified seeds of *Passiflora incarnata*, cultivar CF-01, were obtained through the company Centroflora Group, and registered on the platform of the Ministry of Agriculture, Livestock and Food Supply. Exsiccates were made with reproductive branches of plants obtained through *P*. *incarnata* seeds (CF-01) and deposited at the Herbarium *Irina Delanova Gemtchujnicov (BOTU)*, at Institute of Biosciences, UNESP, under the code BOTU34797. The studies with plants used in this work were carried out in accordance with relevant institutional, national or international guidelines. Propagation occurred by sowing, in December 2018, in a commercial substrate composed of peat, vermiculite, carbonized rice husk and organic residue, using 40 mL tubes. At 81 DAS, the seedlings were transferred to hydroponics. Two plants per pot, with a 6 L capacity, were kept in nutrient solution number 2 by Hoagland and Arnon^39^, at 25% ionic strength, which was raised to 50% at 96 DAS.

### Experimental design and treatments application

The experiment was installed in a randomized block design, with five replications and two plants per repetition. A 4 × 2 factorial design was used, with silicon dioxide (SiO_2_) concentrations equal to 0, 1, 2 and 3 mM and the presence or absence of mechanical damage. At 124 DAS, SiO_2_ was added to the solution and at 136 DAS mechanical damage was done to the main branch, by removing the aerial part of one of the plants (leaving other intact), 15 cm from the pot surface, maintaining a tiller. The preparation of 1 L of SiO_2_ 1 M stock solution was prepared under stirring and heating at 80 °C, with the addition of 0.3 L of NaOH 1 M for its solubilization. When the Si was supplied to the nutrient solution, hydrochloric acid was used to adjust the pH, which was kept between 5.5 and 6.5.

### Measurement of chlorophyll a fluorescence and gas exchange

Chlorophyll a fluorescence and gas exchange were evaluated at 140 and 169 DAS, using the Infra-Red Gas Analyzer, model GFS-3000 Fl-Walz, with a coupled portable modulated light fluorometer. The evaluations took place between 9 a.m. and 11 a.m. on a fully expanded leaf.

The variables evaluated were potential quantum efficiency of open PSII center (Fv′/Fm′) energy fraction absorbed by PSII antenna that is dissipated as heat (*D*), energy fraction not dissipated in the antenna that cannot be used for photochemistry stage (*Ex*), photochemical quenching (qL), electron transport rate (ETR), effective quantum efficiency of photosystem II (ΦPSII), CO_2_ assimilation rate (*A*_*net*_, μmol CO_2_ m^−2^ s^−1^), transpiration rate (*E,* mmol H_2_O m^−2^ s^−1^), stomatal conductance (*g*_*s*_, mmol m^−2^ s^−1^), and Ribulose 1,5-diphosphate carboxylase/oxygenase (RuBisCO) carboxylation efficiency, by the CO_2_ assimilation rate and internal CO_2_ concentration in the sub-stomatal chamber (*A*_*net*_/*C*_*i*_ μmol m^−2^ s^−1^ Pa^−1^).

### Plant material samples for biochemical analysis, vitexin and leaf silicon content

At 169 DAS leaves were collected and frozen in liquid nitrogen to determine carbohydrates, H_2_O_2_ and lipid peroxidation. Part of the collected leaves were dried at 38 °C in a forced ventilation oven to determine vitexin and leaf Si content.

### Determination of total sugars, reducing sugars, sucrose and starch

The total soluble sugars were obtained by triple extraction, with 80% ethanol and supernatants were combined. The pellet from this stage was frozen for subsequent extraction of starch^40^. Them, starch was extracted by triple extraction with chilled 52% perchloric acid and the supernatants were pooled in falcon until reading.

The quantification of total soluble sugars was performed using the anthrone method, with a spectrophotometer reading at 620 nm, expressed in a standard glucose curve^41,42^. Reducing sugars were quantified with the use of dinitrosalicylic acid (DNS), with a reading at 540 nm and a curve expressed in a glucose pattern^43^. Sucrose quantification occurred with the use of an anthrone + 30% KOH, with 620 nm reading and curve expressed in a sucrose pattern^44^. The starch was determined by the anthrone method, and the reading occurred at 620 nm, with a glucose pattern curve.

### Determination of hydrogen peroxide and lipid peroxidation

H_2_O_2_ content was determined with trichloroacetic acid (TCA) and reading on a spectrophotometer at 390 nm^45^. Lipid peroxidation was determined with thiobarbituric acid (TBA) and trichloroacetic acid (TCA) and expressed by the formation of malonaldehyde (MDA)^46^.

### Determination of vitexin and polyphenols

Determination of vitexin according to Wosch et al.^47^, used 200 mg of crushed dry leaves (38 °C), with the addition of 8 mL of 60% ethanol in 15 mL test tubes. Then, the tubes were vortexed (15 s) and submitted to an ultrasound bath (30 min). Each extract was filtered with cotton and the volume was made up with solvent extractor (ethanol). Samples were filtered with a Millex LCR filter (non-sterile 0.45 μm 13 mm PTFE membrane) and placed in amber glass bottles at 4 °C. The quantification of vitexin in the samples was performed in a High-Performance Liquid Chromatography (uv HPLC focused Thermo Fisher-Scientific) with gradient pump and UVVIS detector and a C18 reverse phase column (150 × 4.6 mm and 5 μm particle diameter). The mobile phase consisted of a 0.5% gradient acetic acid in ultrapure water (A), methanol (B) and acetonitrile (C), HPLC grade, with a flow rate of 1 mL/min for 30 min and detection wavelength 340 nm. The standard used was Sulpleco, degree of purity ≥ 95% and the linearity curve of vitexin showed a correlation coefficient, y = 0.8233x − 3.9105, R^2^ = 0.9959. Polyphenols were evaluated by Dualex Scientific optical sensor at 375 nm^48^, at 165 DAS, on both sides of the second and third leaves of each plant fully expanded, avoiding the central rib.

### Leaf silicon content

Leaf Si content was determined using hydrochloric acid and ammonium molybdate, with a reading at 410 nm^49^.

### Growth parameters measurement

Root, stem and leaves of the intact plant were collected at 169 DAS and after determining the leaf area, by leaf area integrator LI-3100C area meter LI-COR, they were subjected to drying at 38 °C until constant dry mass.Growth rates were evaluated by masses averages of dry roots (RDM), stems (SDM), leaves (LDM), aerial part (APDM) and total (TDM). The leaf area (LA) was also evaluated. From these data, leaf mass ratio (LMR) was calculated using the LDM/TDM ratio, and specific leaf area (SLA), LA/LDM^50^.

### Statistical analysis

The results were subjected to variance analysis at 5% significance. The averages were compared by the Tukey test (5% significance).

A heatmap was prepared from the hierarchical cluster analysis, performed using the online version of MetaboAnalyst 4.0^51^, to assess the relationship between treatments and biochemical variables. Variables were standardized and the Euclidean distance between treatments was considered.
